# Downregulating *Nrl* Expression and Rod Photoreceptor Protection

**DOI:** 10.3390/ijms27114683

**Published:** 2026-05-22

**Authors:** Yiwen Li, Shuliang Jiao, Weng Tao, Rong Wen

**Affiliations:** 1 Bascom Palmer Eye Institute, Department of Ophthalmology, University of Miami, Miller School of Medicine, Miami, FL 33136, USA; yiwenxyz@gmail.com; 2 Department of Biomedical Engineering, Florida International University, Miami, FL 33174, USA; shjiao@fiu.edu; 3 QOL Medical, LLC, Vero Beach, FL 32963, USA; wengtao4@gmail.com

**Keywords:** hereditary retinal degeneration, retinitis pigmentosa, photoreceptor reprogramming, *Nrl*, *Nrl* downregulation, shRNA, mouse

## Abstract

Retinitis pigmentosa (RP) is a genetically heterogeneous group of inherited retinal degenerations with primary degeneration of rod photoreceptors followed by secondary cone loss. We investigated whether downregulating Nrl (neural retina leucine zipper), a key transcription factor specifying rod fate, can reprogram rods into a more resilient state. In a transgenic *Nrl^N/N^* mouse in which *Nrl* was markedly downregulated, the rod phenotype became more like a rod precursor, particularly in the inferior retina. Crossing *Nrl^N/N^* mice with two rod degeneration models, *rd1* (*Pde6b^rd1/rd1^*) and rhodopsin P23H knock-in (*Rho^P23H/P23H^*) mice, showed significantly improved photoreceptor survival in double-mutant mice. In addition, AAV-mediated delivery of shRNA targeting *Nrl* mRNA substantially enhanced photoreceptor survival in *rd10* (*Pde6b^rd10/rd10^*) mice. These findings demonstrate that downregulation of *Nrl* reprograms rods and confers broad resistance to degeneration across multiple RP models. AAV-mediated *Nrl* knockdown represents a promising mutation-independent therapeutic strategy for autosomal recessive and dominant forms of RP.

## 1. Introduction

Retinitis pigmentosa (RP) comprises a clinically and genetically heterogeneous group of inherited retinal dystrophies affecting more than 1.5 million patients worldwide [1]. The disease is characterized by primary degeneration of rod photoreceptors, followed by secondary cone loss. RP patients experience night blindness (nyctalopia) and progressive constriction of the peripheral visual field. The decrease in visual field is exponential at 2.6–13.5% per year in the remaining visual-field area, ultimately resulting in tunnel vision and total blindness [2,3]. To date, pathogenic variants in more than 100 genes have been implicated in RP [4]. Despite significant research progress, no approved disease-modifying therapy exists to halt or slow the photoreceptor degeneration.

Rod photoreceptors differentiate from rod precursor cells, S-cones. *Nrl* (neural retina leucine zipper) is a master transcription factor that not only controls rod differentiation but also maintains rod phenotype [5]. In humans, autosomal recessive *Nrl* mutations are associated with retinal dystrophy [6], and a loss-of-function mutation causes enhanced S-cone syndrome [7]. Also, autosomal dominant variants of *Nrl* Nr are linked to an autosomal dominant retinal degeneration known as RP 27 [8,9].

Since rods are the target of RP-related retinal degeneration, a strategy to protect rods is to alter the phenotype of rods by reprogramming rod photoreceptors. *Nrl* has been the focus of such research. Studies showed that genetic knockout of *Nrl* prevents rod precursors from differentiating into rod photoreceptors, and the cells remain as S-cones [10,11,12]. Matured rods in adult animals could also be reprogrammed by conditional knockout or gene editing to neutralize the *Nrl* gene, and the reprogrammed cells were resistant to degeneration [13,14,15,16].

The studies mentioned above, neutralizing the *Nrl* gene by conditional knockout and in vivo gene editing, demonstrated that manipulating the *Nrl* gene could reprogram rods and make them resistant to degeneration, and provided the proof of concept for targeting *Nrl* as a promising therapeutic avenue for RP [17]. The technical challenge is how to knock out *Nrl* in human rod cells. Conditional knockout of *Nrl* is obviously not practical for direct clinical treatment in human patients, as it requires complex, multi-step engineering, including homologous recombination in embryonic stem cells to insert recombinase recognition sites (e.g., loxP sites for Cre recombinase) flanking the target sequence [18]. To develop *Nrl* targeting as a clinically viable therapy requires a major technical breakthrough to inhibit *Nrl* expression safely and effectively in the adult human retina.

We hypothesized that downregulating the expression of *Nrl* could reprogram rod photoreceptors and protect them from degeneration without gene knockout. In the present work, we show that downregulating *Nrl* protects photoreceptors from degeneration. Reprogramming rod photoreceptors was demonstrated in a transgenic *Nrl^N/N^* mouse line in which *Nrl* expression was markedly reduced. When *Nrl^N/N^* mice were crossed with two mouse models of RP, *rd1* (*Pde6b^rd1/rd1^*) and rhodopsin P23H knock-in (*Rho^P23H/P23H^*) mice, photoreceptor survival was significantly enhanced in the double-mutant mice. We further investigated downregulating *Nrl* with short hairpin RNA (shRNA) targeting *Nrl* mRNA. Adeno-associated virus (AAV)-delivered shRNA conferred significant photoreceptor protection against degeneration in *rd10* (*Pde6b^rd10/rd10^*) mice. The present work represents a major technical advance and establishes *Nrl* downregulation as a promising mutation-independent therapeutic strategy for autosomal dominant and recessive RP.

## 2. Results

### 2.1. Downregulation of Nrl Expression in Nrl^N/N^ Mice

**Figure 1:**
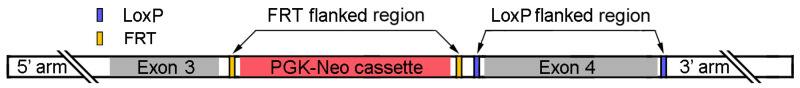
A schematic of the *Nrl^N^* DNA construct. The *PGK-Neo* cassette (red), flanked by two FRT sites (yellow), was inserted into the intron between exons 3 and 4 (gray) of the mouse *Nrl* gene. The *PGK-Neo* cassette is removable in the presence of recombinase flippase (FLP). Exon 4 is flanked by two loxP sites (blue).

**Figure 2:**
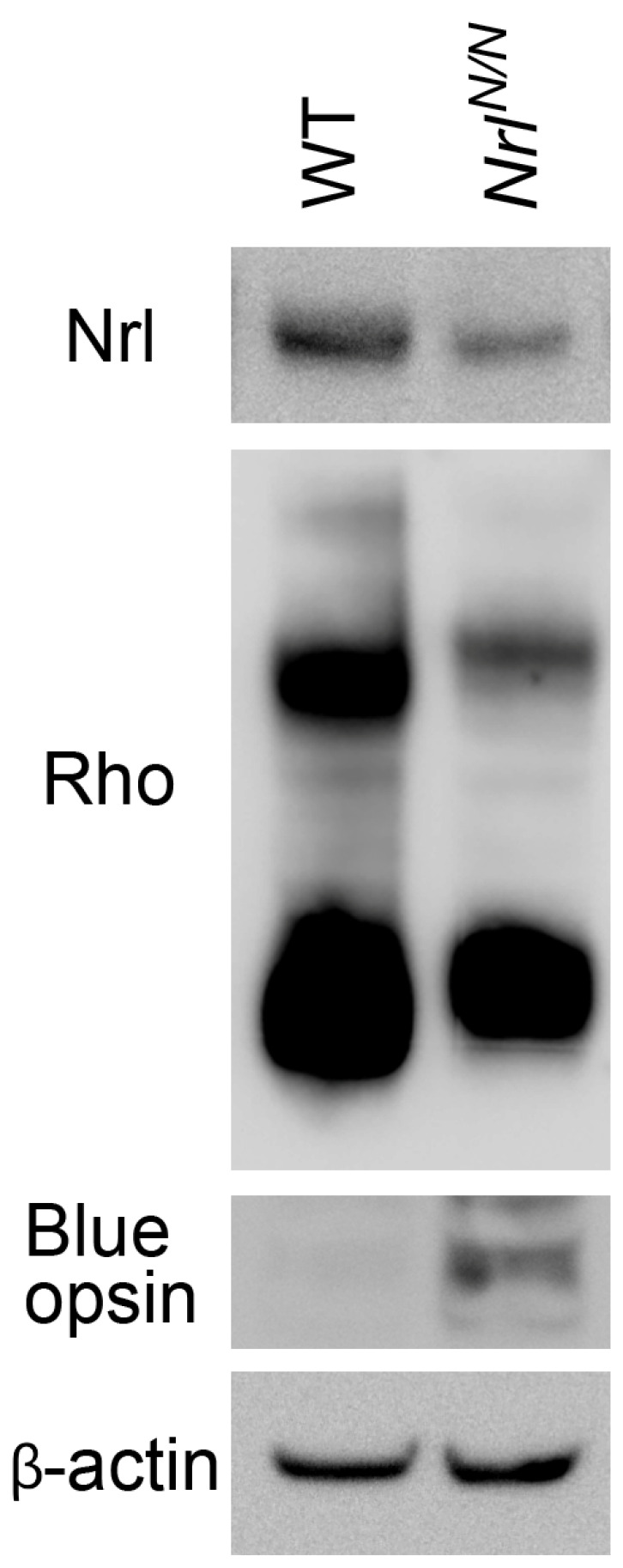
*Nrl* downregulation in *Nrl^N/N^* mice. Retinas were collected at PD (postnatal day) 60 from *Nrl^N/N^* and WT (wildtype C57) mice. Protein levels were examined by Western blot analysis. In *Nrl^N/N^* mice, the level of *Nrl* was significantly reduced compared with that in the WT control. The levels of rhodopsin (Rho) were also reduced, whereas the level of S-opsin was increased in *Nrl^N/N^* mice. The levels of β-actin served as loading controls.

A transgenic *Nrl^N/N^* mouse line was generated by inserting a *PGK-Neo* cassette flanked with two FRT sites (*Nrl^N^*) into the intron between exons 3 and 4 of the *Nrl* locus (Figure 1). The *PGK-Neo* cassette is known to influence the expression of genes [19,20]. Mice homozygous for the *Nrl^N^* allele are viable, fertile, and grossly normal. Western blot analysis showed that *Nrl* protein in retinas of *Nrl^N/N^* mice was significantly reduced (Figure 2), accompanied by an increase in S-opsin and a decrease in rhodopsin protein (Figure 2). These results confirmed that *Nrl* expression was downregulated in *Nrl^N/N^* mice, and rod differentiation was attenuated.

**Figure 3:**
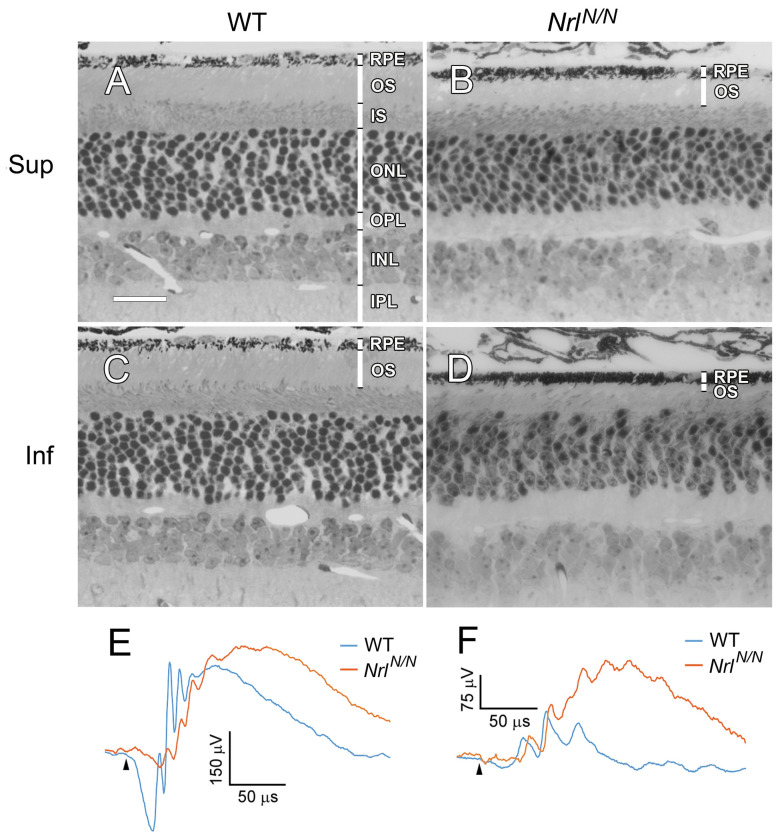
Retinal morphology and ERG of *Nrl^N/N^* mice. Eyes were collected from PD 60 *Nrl^N/N^* and WT mice. The retina of the *Nrl^N/N^* mouse had normal layers in laminar organization (**B**,**D**) compared with that of the WT mouse (**A**,**C**), with 1 row of nuclei in the ONL thinner than the WT. The rod outer segments (OSs) were shorter in the *Nrl^N/N^* mouse (**B**,**D**) than in the control retina (**A**,**C**), especially in the inferior retina (**D**). At PD 60, the dark-adapted ERG from *Nrl^N/N^* mouse had a very small a-wave, and the b-wave amplitude was smaller than that of the WT mouse ((**E**); elicited by −0.402 log cd·s/m^2^ white flashes). The light-adapted b-wave from *Nrl^N/N^* mice was larger than that from WT mice ((**F**); elicited by 0.998 log cd·s/m^2^ flashes with 30 cd/m^2^ white background). Black arrowheads in (**E**,**F**) indicate ERG flash onset. Retinal layers are indicated by white bars in panel A. RPE: retinal pigment epithelium; OS: outer segment; IS: inner segment; ONL: outer nuclear layer; OPL: outer plexiform layer; INL: inner nuclear layer; IPL: inner plexiform layer. Sup: superior; Inf: inferior. Scale bar: 25 μm.

Morphologically, the retinas of *Nrl^N/N^* animals (Figure 3B,D) exhibited normal retinal layers with normal laminar organization compared with the retinas of WT mice (Figure 3A,C), although in the *Nrl^N/N^* mice, the ONL (outer nuclear layer) was one row of nuclei less than that of the wild-type mice. Photoreceptor outer segments (OSs) were shorter in *Nrl^N/N^* mice (Figure 3B,D), especially in the inferior retina (Figure 3D). No retinal disorganization or rosette was observed in *Nrl^N/N^* mice (Figure 3B,D), unlike in *Nrl*-knockout mice, in which retinal disorganization and rosette are common [10].

Dark-adapted ERGs (electroretinograms) from *Nrl^N/N^* mice showed a very small a-wave, consistent with short photoreceptor outer segments (Figure 3E). The b-wave in the dark-adapted ERG was smaller than that from the WT mice (Figure 3E). In the light-adapted ERG, the b-wave from *Nrl^N/N^* mice was larger than that from WT mice, consistent with more cone-like cells (Figure 3F, see below).

**Figure 4:**
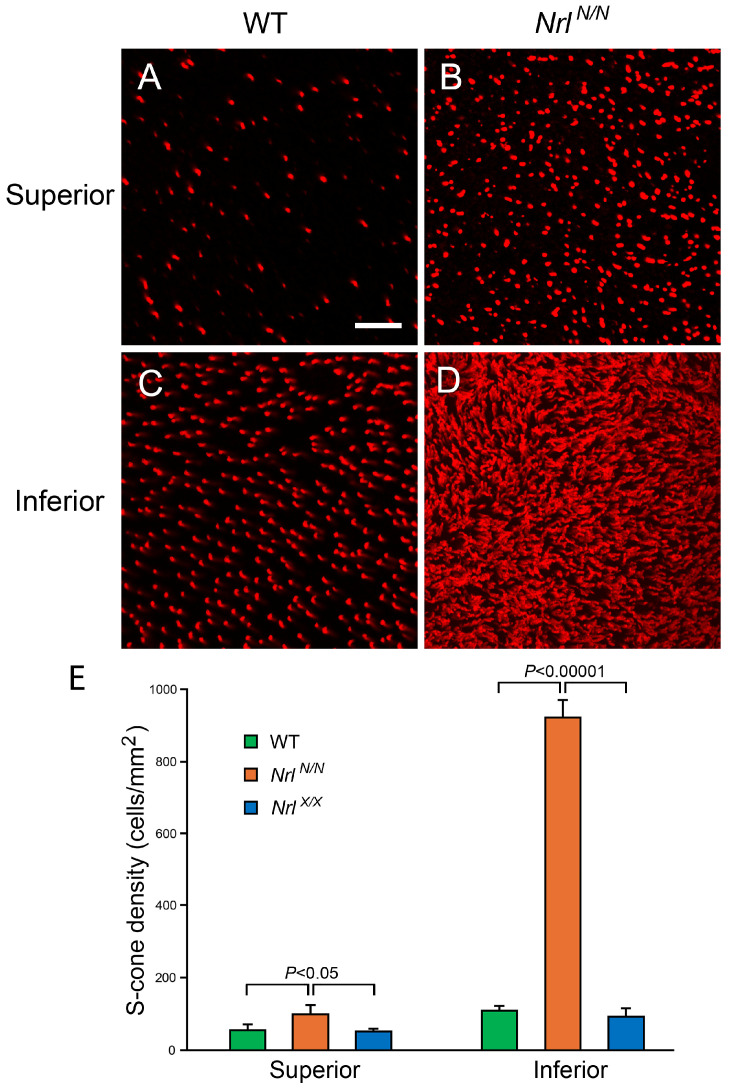
S-opsin-positive cells in the retina. Eyes were collected at PD 60 from *Nrl^N/N^* and WT mice. Immunocytochemical analysis in flat-mounted retinas showed many S-opsin-positive cells in the retina of the *Nrl^N/N^* mouse (**B**,**D**), especially in the inferior retina (**D**), compared with the superior (**A**) and inferior retina (**C**) of the WT (wild-type) mouse. The average density of S-opsin-positive cells in the superior retinas of *Nrl^N/N^* mice was significantly higher than that in either WT mice (*p* < 0.05; *n* = 3) or *Nrl^X/X^*mice in which the *PGK-Neo^r^* cassette was removed (*p* < 0.05; *n* = 3) (**E**). In the inferior retina, the density of S-opsin-positive cells in *Nrl^N/N^* mice was much higher than in the WT and *Nrl^X/X -N/-N^* mice (*p* < 0.0001; *n* = 3) (**E**). The S-opsin-positive cell densities in the *Nrl^X/X^* mouse in the superior or the inferior retina were comparable to those in the WT mouse (**E**). In addition, the density of S-opsin-positive cells in the inferior retina in WT mice was significantly higher than in the superior retina (*p* < 0.005; *n* = 3), as well as in *Nrl^N/N^* mice (*p* < 0.0001; *n* = 3) and *Nrl^X/X^* mice (*p* < 0.05; *n* = 3). Sup: superior; Inf: inferior. Scale bar: 20 µm.

*Nrl^N/N^* animals had significantly more S-opsin-positive cells in the retina (Figure 4B,D) than in wild-type mice (Figure 4A,C), especially in the inferior retina (Figure 4D). The average density of S-opsin-positive cells in the inferior retinas of *Nrl^N/N^* mice was 8.3 times the density in the inferior retinas of WT mice (Figure 4E; Table 1). When the *PGK-Neo* cassette was removed from *Nrl^N/N^* mice by crossing them with *R26^FLPe^* mice, the density of S-opsin-positive cells in the *Nrl ^X/X^* mice was not significantly different from that in WT retinas (Figure 4E). *R26^FLPe^* mice express FLPe (an enhanced version of flippase) [21] in multiple organs, including the retina.

These results indicate that downregulation of *Nrl* expression reprograms rod photoreceptors, and cells in the inferior retina are more sensitive to *Nrl* downregulation than cells in the superior retina.

It is worth noticing that the density of S-opsin-positive cells in the inferior retinas in WT mice was significantly higher than that in the superior retinas (Figure 4E; Table 1). The densities of S-opsin-positive cells were higher in the inferior retinas in *Nrl^N/N^* and *Nrl^X/X^* mice (Figure 4E; Table 1).

### 2.2. Nrl Downregulation and Photoreceptor Survival

To investigate the effects of *Nrl* downregulation on photoreceptor degeneration, we crossed *Nrl^N/N^* mice with two rod degeneration models: the *rd1* (*Pde6b^rd1/rd1^*) mouse and the *Rho* P23H knock-in mouse (*Rho^P23H/P23H^*). The *Rd1* mouse carries the *rd1* mutation in the *Pde6b* gene and is a model widely used in RP research [22]. The *Rho^P23H/P23H^* mouse is a transgenic mouse harboring the P23H mutation in the *Rho* gene [23]. The *Rho* P23H mutation is the most common mutation in autosomal dominant RP patients [3,23].

**Figure 5:**
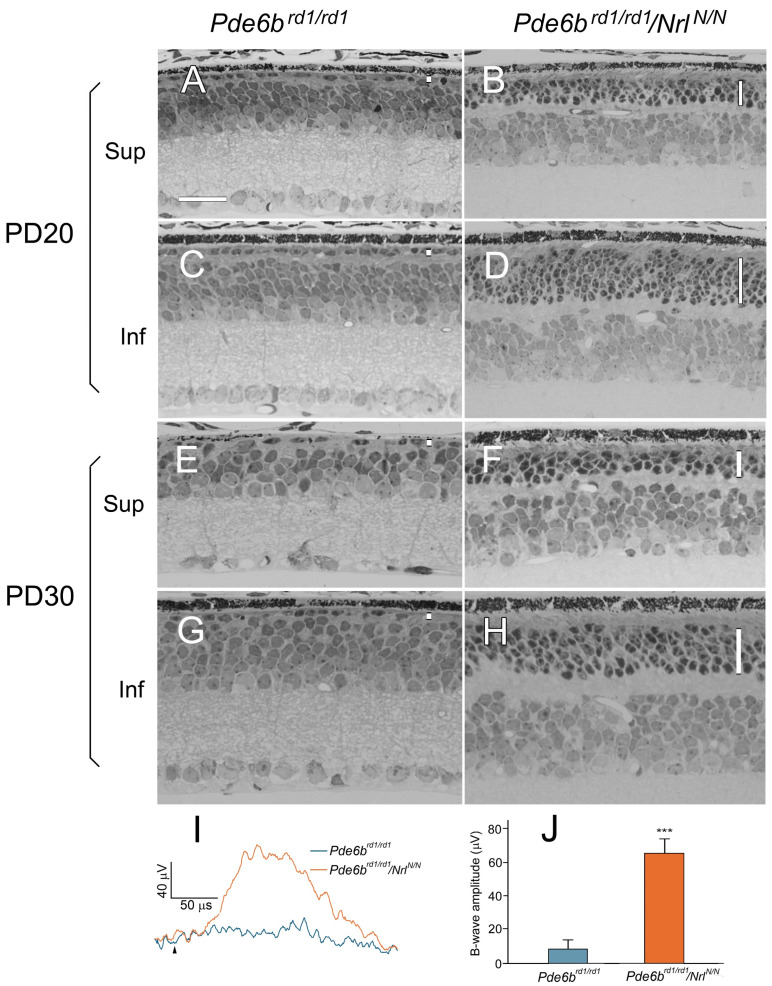
Photoreceptor preservation in mice with Pde6b^rd1/rd1^/*Nrl^N/N^*double mutants. Retinal degeneration in the *rd1* (Pde6b^rd1/rd1^) mouse is rapid. The ONL had 1 row of nuclei at PD 20 in both the superior (**A**) and inferior retina (**C**), and less than 1 row of nuclei by PD 30 (**E**,**G**). In the Pde6b^rd1/rd1^/*Nrl^N/N^* mouse, photoreceptors were well-preserved. At PD 20, the ONL had 3–4 rows of nuclei in the superior retina, and 5–6 rows in the inferior retina (**B**,**D**), and by PD 30, the ONL still had 3 rows in the superior retina, and 5 rows in the inferior retina (**F**,**H**). The ERG (elicited by 0.398 log cd·s/m^2^ white flashes with a 30 cd/m^2^ white background) from the Pde6b^rd1/rd1^ mouse was flat, whereas the b-wave from the Pde6b^rd1/rd1^/*Nrl^N/N^* mouse was significantly larger at PD 30 ((**I**,**J**); *** *p* < 0.0001; *n* = 3). Black arrowhead indicates flash onset. The ONL in each retinal section is indicated by a vertical white bar (**A**–**D**). Sup: superior; Inf: inferior. Scale bar: 25 µm.

Photoreceptors in *rd1* mice underwent rapid degeneration (Figure 5A,C,E,G). The ONL had one row of nuclei at PD 20 (postnatal day 20) (Figure 5A,C) and less than one row by PD 30 (Figure 5E,G). In double-mutant *Pde6b^rd1/rd1^*/*Nrl^N/N^* mice, the ONL had 3–4 rows of nuclei in the superior retina and 5–6 rows in the inferior retina at PD 20 (Figure 5B,D). At PD 30, the ONL in the superior retina had three rows of photoreceptor nuclei in the ONL and five in the inferior retina (Figure 5F,H). The ERG from the *rd1* mouse was flat at PD 30, whereas the ERG b-wave from the *Pde6b^rd1/rd1^*/*Nrl^N/N^* mouse was significantly larger (Figure 5I,J).

**Figure 6:**
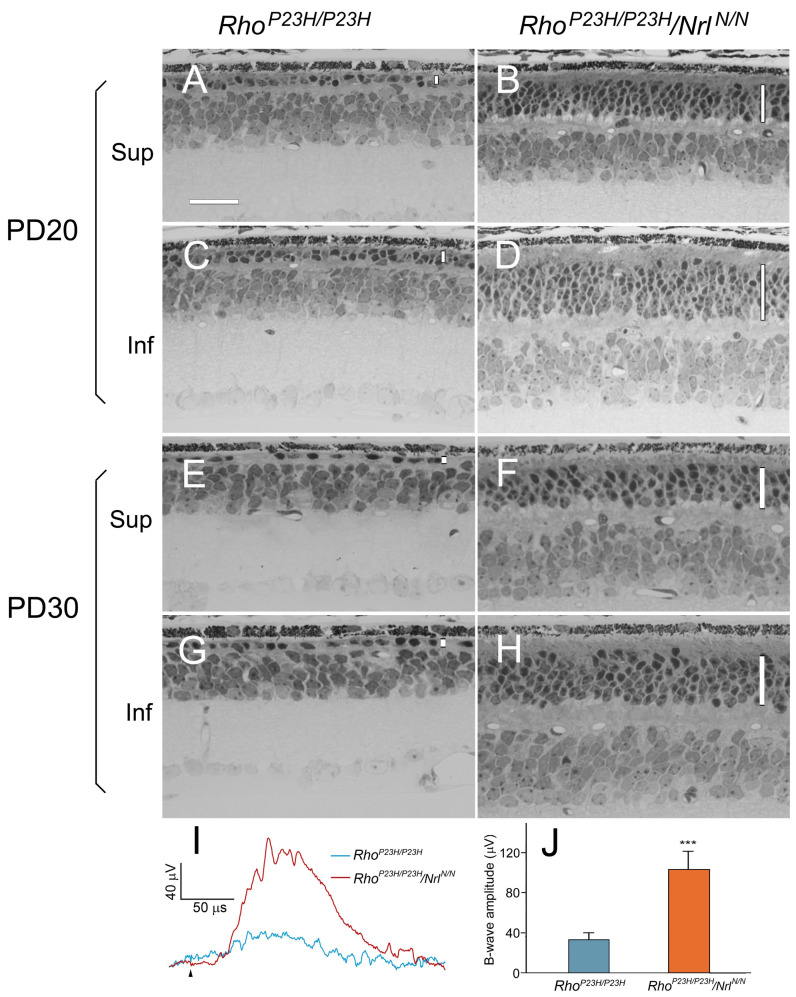
Photoreceptor preservation in mice with *Rho^P23H/P23H^*/*Nrl^N/N^* double mutants. Retinal degeneration in *Rho^P23H/P23H^* mouse was so rapid that the ONL had less than 1–2 rows of nuclei in the superior retina (**A**) and 2 rows of nuclei in the inferior retina (**C**) at PD 20, and by PD 30, the ONL had less than 1 row of nuclei in the superior retina (**E**) and 1 row in the inferior retina (**G**). In the *Rho^P23H/P23H^*/*Nrl^N/N^* mouse, the ONL had 4 rows of nuclei in the superior retina (**B**) and 6 rows in inferior retina (**D**) at P20. By PD 30, the ONL still had 3–4 rows of nuclei in the superior retina (**F**) and 5–6 rows in the inferior retina (**H**). The ERG at PD 30, elicited by 0.398 log cd·s/m^2^ white flashes with a 30 cd·s/m^2^ white background, showed that the b-wave from the *Rho^P23H/P23H^* mouse was very small, but the b-wave from the Pde6b^rd1/rd1^/*Nrl^N/N^* mouse was significantly larger ((**I**,**J**); *** *p* < 0.001; *n* = 3). Black arrowhead indicates flash onset. ONL in each retinal section is indicated by a vertical white bar (**A**–**D**). Sup: superior; Inf: inferior. Scale bar: 25 µm.

Retinal degeneration was also fast in *Rho^P23H/P23H^* mice. At PD 20, the ONL had 1–2 rows of nuclei in the superior retina and two rows in the inferior retina (Figure 6A,C). By PD 30, the ONL had less than one row of nuclei in the superior retina and one row in the inferior retina (Figure 6E,G). In *Rho^P23H/P23H^*/*Nrl^N/N^* mice, however, the ONL had four rows of photoreceptor nuclei in the superior retina (Figure 6B) and six rows in the inferior retina at P20 (Figure 6D). And by PD 30, the ONL still had 3–4 rows of nuclei in the superior retina (Figure 6F) and 5–6 rows in the inferior retina (Figure 6H). The ERG from the *Rho^P23H/P23H^* mouse was almost flat at PD 30. In contrast, the ERG b-wave from the *Rho^P23H/P23H^*/*Nrl^N/N^* mouse was significantly larger (Figure 6I,J).

These results indicate that reprogrammed photoreceptors are resistant to RP-associated degeneration.

### 2.3. Nrl Downregulation with shRNA Targeting Nrl mRNA

We next assessed the downregulation of *Nrl* expression by shRNA. ShRNA targeting different regions of the ORF (open reading frame) of mouse *Nrl* mRNA were predicted by two online algorithms (see Materials and Methods). Sequences of shRNA that effectively downregulated *Nrl* expression were selected, and a full shRNA was created by inserting an shRNA-targeting sequence into the mirE backbone [24] and fused to the 3′ UTR (untranslated region) of a small fluorescent protein, CagFbFP [25].

**Figure 7:**
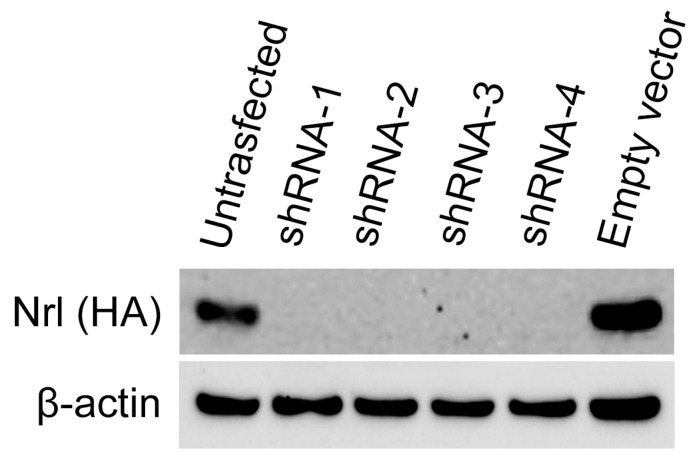
*Nrl* downregulation by shRNA. 293-Nrl cells stably expressing mouse *Nrl* were used to evaluate the capability of downregulating *Nrl* expression by shRNA. Cells were transfected with a plasmid containing a given shRNA and harvested 72 h after transfection. Western blots showed that 4 shRNAs successfully downregulated *Nrl* expression. Untransfected 293-Nrl cells and 293-Nrl cells transfected with the empty vector served as controls. The levels of β-actin in each sample served as loading controls.

The capability of a given shRNA to downregulate *Nrl* expression was evaluated in 293-Nrl cells that stably overexpressed mouse *Nrl*. A plasmid containing a given shRNA* (pRVS-CagFbFP-shRNA*) was transfected into 293-Nrl cells, and the transfected cells were harvested 72 h later. Western blot analysis showed *Nrl* expression was blocked by each shRNA effectively (Figure 7). shRNA not targeting mouse *Nrl* had no effects on *Nrl* expression.

Two shRNAs (shRNA-2 and -4) shown in Figure 7 were selected and packaged in AAV for experiments with *rd10* mice, a retinal degeneration model for experimental therapy [22]. The right eye of an *rd10* mouse was injected with 1.5 µL of either AAV-shRNA-2 or AAV-shRNA-4 into the subretinal space at PD 14, and the left eye was injected with 1.5 µL of control AAV-GFP. Eyes were collected by PD 35 for morphological analysis. ERGs were recorded before eye collection.

**Figure 8:**
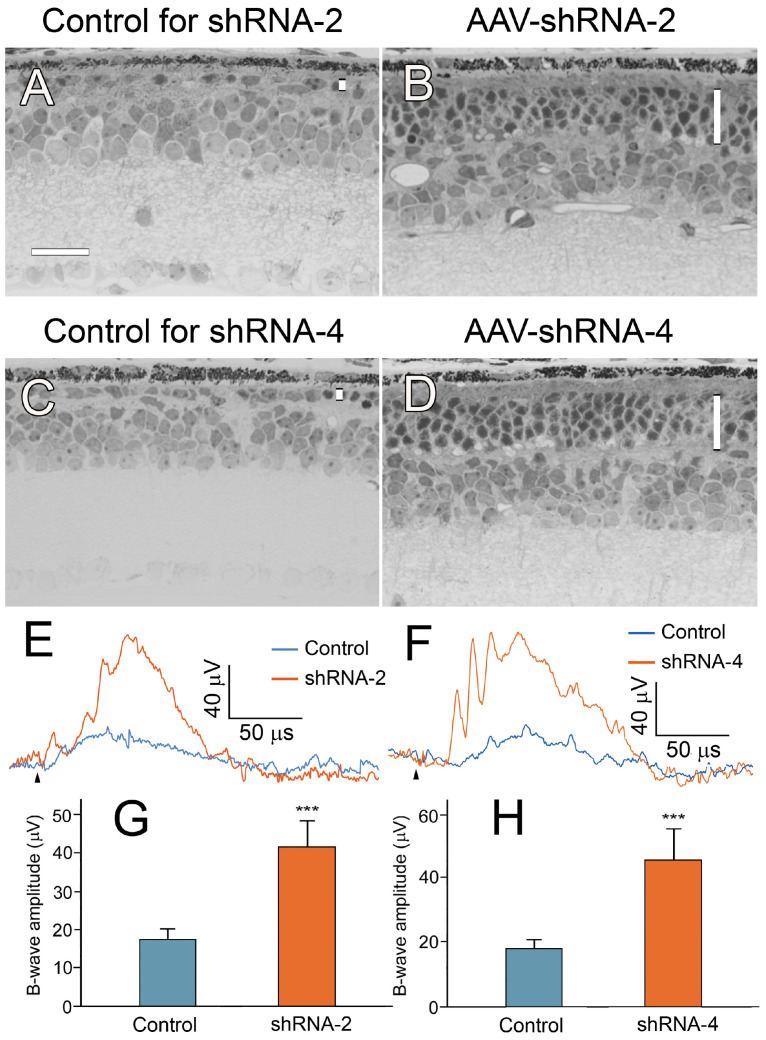
Photoreceptor protection by shRNA in mice with *Pde6b^rd10/rd10^*mutation. The right eyes of *rd*10 mice were injected with AAV-shRNA-2 or AAV-shRNA-4 into the subretinal space at PD 14, and the left eyes were injected with AAV-GFP as injection controls. Eyes were collected at PD 35. The retinas in the control eyes had 1 row of nuclei in the ONL (**A**,**C**). In the eyes injected with AAV-shRNA-2 (**B**) or AAV-shRNA-4 (**D**), the ONL had 4 rows of nuclei in the injected area (**B**,**D**). The ERG b-waves from the control eyes were very small (**E**,**F**), but the b-waves from eyes injected with AAV-shRNA-2 (**E**,**G**) or AAV-shRNA-4 (**F**,**H**) were significantly larger (*** *p* < 0.001; *n* = 3 for (**G**,**H**)) (ERGs were elicited by 0.398 log cd·s/m^2^ white flashes with a 30 cd/m^2^ white background). Black arrowheads indicate flash onset. The ONL in each retinal section is indicated by a vertical white bar (**A**–**D**). Scale bar: 25 µm.

In the eyes treated with AAV-shRNA-2 (Figure 8B) or AAV-shRNA-4 (Figure 8D), the ONL in the injected area had 4–5 rows of nuclei, compared with one row of nuclei in the control eyes (Figure 8A,C). The ERG b-wave amplitudes from the eyes injected with either AAV-shRNA-2 or AAV-shRNA-4 were larger than those from control eyes (Figure 8E–H).

## 3. Discussion

We showed that the rod photoreceptor phenotype can be reprogrammed by downregulating *Nrl* expression. In the transgenic *Nrl^N/N^* mouse, *Nrl* expression was effectively downregulated by a *PGK-Neo* cassette inserted into the intron between exons 3 and 4 of the *Nrl* gene. The *PGK-Neo* cassette is known to downregulate nearby genes [19,20].

The most important finding from experiments with the *Nrl^N/N^* mouse is that rod photoreceptors can be reprogrammed by reducing *Nrl* expression, indicating that the function of *Nrl* is a graded regulator of rod phenotype rather than an all-or-none determinant of rod differentiation, as previously assumed. The discovery of reprogramming rod photoreceptors by reducing *Nrl* expression also represents a major advance toward developing mutation-independent therapies for RP.

Targeting *Nrl* by genomically neutralizing the *Nrl* gene, as shown by previous studies [13,14,15,16], poses substantial hurdles for clinical translation. In contrast, downregulation of *Nrl* expression is technically straightforward and clinically feasible. Our results showed that shRNA-mediated suppression of *Nrl* mRNA effectively enhanced photoreceptor survival when delivered by an AAV. These findings provide compelling preclinical evidence for an AAV-shRNA-based, mutation-independent therapeutic strategy for RP.

shRNA is a powerful tool for knocking down gene expression by targeting specific mRNA sequences. A typical shRNA consists of a target-specific stem and a loop, with the full shRNA sequence usually under 100 bp. In the present work, we designed a compact all-in-one expression cassette (<2.5 kb) that includes a tissue-specific promoter, the ORF of a small fluorescent reporter (CagFbFP), the shRNA sequence embedded in the 3′ UTR, and flanking AAV inverted terminal repeats (ITRs). The size was small enough to be packaged into a double-stranded AAV (dsAAV) vector [26]. Unlike a single-stranded AAV (ssAAV) that requires rate-limiting second-strand synthesis in host cells, a dsAAV delivers a transcription-ready double-stranded genome, resulting in a faster onset and higher transgene expression [26]. Moreover, the small footprint of each shRNA allows multiple shRNAs targeting different regions of the same mRNA to be combined within a single vector, thereby enhancing the overall knockdown efficiency.

A striking feature of the *Nrl^N/N^* mouse was the marked dorsoventral difference in rod photoreceptor differentiation. Photoreceptor outer segments in the inferior retina were substantially shorter (Figure 3), and the number of S-opsin-positive cells was ~9-fold higher than in the superior retina (Figure 4; Table 1). Thus, rods in the inferior retina were considerably more sensitive to reduced *Nrl* expression than those in the superior retina. These findings indicate that rods in the inferior retina are less differentiated than those in the superior retina in *Nrl^N/N^* mice. In wild-type mice, the density of S-opsin-positive cells in the inferior retina was also significantly higher than that in the superior retina (Figure 4, Table 1), suggesting similar dorsoventral asymmetry in rod differentiation.

It is surprising to notice that rods in the superior retina were well-preserved in both *Pde6b^rd1/rd1^*/*Nrl^N/N^* (Figure 5B) and *Rho^P23H/P23H^*/*Nrl^N/N^* mice (Figure 6B), even though rods in this region showed limited reprogramming in the *Nrl^N/N^* background (as discussed above). Thus, a substantial increase in rod survival was achieved with a low level of rod reprogramming, suggesting that therapeutic strategies aimed at downregulating *Nrl* for RP could have a broad therapeutic dose window. A successful clinical outcome may not require complete or a high level of *Nrl* suppression.

Our results from *Nrl^N/N^* mice indicate that *Nrl* is essential for maintaining normal retinal layers and laminal organization, even at a low level. Retinal disorganization and rosette formation are common in *Nrl*-knockout (*Nrl^-/-^*) mice when *Nrl* expression is absent [10,13]. No such structural disruption or retinal disorganization was observed in *Nrl^N/N^* mice (Figure 3A). When considering targeting *Nrl* as a therapy for RP, downregulating *Nrl* expression is likely a safer approach than gene knockout, as the latter may carry the risk of inducing retinal disorganization and rosette formation as unintended side effects.

Rod photoreceptor reprogramming would reduce retinal light sensitivity, thereby decreasing overall scotopic vision in treated eyes. In modern environments with ubiquitous artificial lighting, however, this is unlikely to cause meaningful functional difficulty. The benefit of halting the progressive retinal degeneration and preserving high-acuity vision by downregulating *Nrl* should far outweigh the modest trade-off in low-light sensitivity.

In summary, we demonstrated that the rod photoreceptor phenotype can be reprogrammed by downregulating the expression of *Nrl*, and our AAV-shRNA experiments provide pre-clinical evidence supporting AAV-shRNA as a mutation-independent therapeutic approach for both recessive and dominant RP.

## 4. Materials and Methods

### 4.1. Animals

Procedures involving animals were approved by the Institutional Animal Care and Use Committee (IACUC) of the University of Miami, the Miller School of Medicine. All methods were carried out in accordance with relevant guidelines and regulations, including adherence to the ARRIVE (Animal Research: Reporting of In Vivo Experiments) guidelines and the ARVO (Association for Research in Vision and Ophthalmology) Statement for the Use of Animals in Ophthalmic and Vision Research.

C57/6, *Pde6b^rd1^*, *Rho^P23H^*, *Pde6b^rd10^* and *R26^FLPe^* mice were purchased from Jackson Labs (Bar Harbor, ME, USA). The animals were kept in a 12 h light/dark cycle at an in-cage illumination of <50 lux. The room temperature was kept at 20–22 °C.

### 4.2. Transgenic Nrl^N/N^ Mouse Generation

A transgenic *Nrl^N/N^* mouse line was generated in the Transgenic Mouse Facility, the University of Miami School of Medicine. The transgene (*Nrl^N^*) DNA construct contained a *PGK-Neo* cassette flanked by a pair of FRT sites inserted into the intron between exons 3 and 4 of the mouse *Nrl* gene (Figure 1). The DNA construct was electroporated into mouse embryonic stem cells. Correctly targeted cells were selected and used to generate chimeric animals. Transgenic chimeric mice were bred to obtain animals homozygous for the *Nrl^N^* allele.

### 4.3. shRNA Design

Sequences of shRNA targeting different regions of mouse *Nrl* mRNA ORF were predicted by 2 algorithms online, the GPP Web Portal of Broad Institute [27] and the SplashRNA of Memorial Sloan Kettering Cancer Center [28], and tested for their efficacy in downregulating *Nrl* expression. A full shRNA was made by placing a targeting sequence in the optimized mirE backbone [24], which was embedded in the 3′ UTR of a small fluorescent protein, CagFbFP [24,25], to create CagFbFP-shRNA. The DNA construct was cloned into the vector pRc/RSV (Invitrogen, Carlsbad, CA, USA) to create the plasmid pRSV-CagFbFP-shRNA. The plasmid containing a given shRNA was tested for downregulating mouse *Nrl* expression in cells overexpressing mouse *Nrl* (see below).

### 4.4. Nrl-Expressing Cells and shRNA Evaluation

A cell line overexpressing mouse *Nrl* was created for testing the capacity of a given shRNA to downregulate mouse *Nrl* expression. A plasmid (pcDNA-mNrl) expressing mouse *Nrl* was created by subcloning the mouse *Nrl* cDNA sequence (ORF plus a C-terminus HA-tag) into pcDNA3.1-puro (Thermo Fisher Scientific, Waltham, MA, USA) and transfected into HEK293T cells (CRL-3211, ATCC, Manassas, VA, USA) using the jetPRIME transfection kit (Avantor, Radnor, PA, USA). Cells were cultured in DMEM (Dulbecco’s modified Eagle’s medium) with 10% fetal calf serum at 37 °C and maintained at 37 °C in 5% CO_2_/95% air. Stably transfected cells were selected with 1 µg/mL of puromycin. A cell colony expressing a high level of *Nrl* was selected as 293-Nrl and expanded.

To evaluate the *Nrl*-downregulating capability of a given shRNA*, a plasmid containing the shRNA (pRVS-CagFbFP-shRNA*; see above) was transfected into 293-Nrl cells. The level of mouse *Nrl* protein was examined 72 h later. Untransfected 293-Nrl cells and cells transfected with the empty vector served as controls.

### 4.5. AAV-shRNA Construction

AAV-shRNA was created by subcloning the DNA construct of a given shRNA* (CagFbFP-shRNA*) into pscAAV, downstream of the human rhodopsin promoter. AAV-shRNA* was then packaged as double-stranded AAV (pscAAV-shRNA*) into serotype AAV2.7m8 for photoreceptor expression [26,29]. AAV-shRNA and AAV-GFP (>2 × 10^13^ GC/mL in PBS) were produced by Vector Builder (Chicago, IL, USA).

### 4.6. Subretinal Injection

To inject AAV-shRNA, an *rd10* mouse was anesthetized with intraperitoneal injections of ketamine (80 mg/kg) and xylazine (10 mg/kg). AAV-shRNA was injected into the subretinal space *via* a 33-gauge blunt-ended needle connected to a 10 µL microsyringe (Hamilton, Reno, NV, USA) under a surgical microscope. The right eye of the mouse was injected with 1.5 µL of AAV-shRNA, and the left eye was injected with the control viral vector AAV-GFP (1.5 µL).

### 4.7. Western Blotting

Retinas were harvested after animals were euthanized using CO_2_ overdose. Cell samples were collected after transfection. Retinal or cell samples were homogenized in the RIPA lysis buffer, and the total protein concentration of a sample was determined by the BCA (bicinchoninic acid) protein assay (Bio-Rad Labs, Hercules, CA, USA). Western blotting was performed using primary antibodies (anti-Nrl ABN1712, Millipore Sigma, Burlington, MA, USA; anti-HA 26183, Thermo Fisher Scientific, Waltham, MA, USA; anti-β actin sc-47778, Santa Cruz Biotechnology, Santa Cruz, CA, USA; anti-S-opsin AB5407, Millipore Sigma; and anti-rhodopsin, B6-30, gift of Dr. Jeremy Nathans) followed by appropriate secondary antibody conjugated to horseradish peroxidase. Antigen signals were detected with a Chemiluminescent Substrate Kit (Thermo Fisher Scientific, Waltham, MA, USA) and imaged on a luminescent image analyzer (ImageQuant LAS-4000, GE Healthcare Life Sciences, Chicago, IL, USA).

### 4.8. Histology

For histological evaluation of the retinas, animals were euthanized using CO_2_ overdose and immediately perfused with mixed aldehydes, as described previously [30,31]. Eyes were removed, semi-sectioned along the vertical meridian, and embedded in an Epon/Araldite mixture [30,31]. Semi-thin (1 µm thick) sections were cut to display the entire retina along the vertical meridian, or through the injected region, and stained with toluidine blue [30,31]. Retinal sections were examined by light microscopy.

### 4.9. Immunocytochemistry

Immunocytochemical staining was employed to identify S-opsin-positive cells in whole-mount retinas. Tissue samples were prepared as described previously [32]. Briefly, the animals were perfused with PBS after being euthanized using CO_2_ overdose. The eyes were collected, and retina–lens preparations were made by removing the corneas and then the sclera–choroid–RPE. The retina–lens preparations were fixed in 4% paraformaldehyde, washed with PBS, and incubated with primary anti-S-opsin antibodies (anti-S-opsin AB5407, MilliporeSigma) and then secondary antibodies conjugated with Cy3 (Jackson ImmunoResearch, West Grove, PA USA). After antibody incubation, the lenses were removed, and the retinas were flat-mounted photoreceptor-side up on slides. Stained whole-mount retinas were examined by confocal microscopy, and cone densities were quantified.

### 4.10. Electroretinogram (ERG)

ERGs were recorded with a UTAS system (LKC Technologies, Gaithersburg, MD, USA). For dark-adapted ERG, animals were dark-adapted for more than 3 h before recording. Light-adapted ERGs were recorded after animals were light-adapted to room light for >30 min.

An animal was put on the animal holder with a heat pad to maintain body temperature at 37 °C after being anesthetized with intraperitoneal injections of ketamine (80 mg/kg) and xylazine (10 mg/kg). A contact lens electrode was placed on the cornea of each eye after the pupils were dilated with 0.1% atropine and 0.1% phenylephrine HCl. A differential electrode was placed under the skin of the forehead, and a ground electrode under the skin at the base of the tail. Full-field ERGs were elicited by 1 ms white flashes generated by white LEDs in the Ganzfeld sphere. The inter-stimulus intervals were 10 s. Each recording was the average of 10 responses.

### 4.11. Statistical Analysis

Statistical analyses were performed using the Prism 10 (GraphPad Software, Boston, MA, USA). Data were evaluated by Student’s *t*-test for comparisons between two experimental groups or ANOVA (analysis of variance) followed by Tukey comparison among three or more groups.

## Figures and Tables

**Table 1:** Densities of S-opsin-positive cells *.

	WT	*Nrl^N/N^*	*Nrl^X/X^*
Superior	57 ± 11	100 ± 23	52 ± 4
Inferior	111 ± 9	925 ± 45	94 ± 20
*p*-value Inferior vs superior	<0.005	<0.0001	<0.05

* Data are presented as cells per 0.006 mm^2^; mean ± SD; *n* = 3.

## Data Availability

The data are presented in this article.

## Abbreviations

The following abbreviations are used in this manuscript:

RP	Retinitis pigmentosa
AAV	Adeno-associated virus
dsAAV	Double-stranded adeno-associated virus
ssAAV	Single-stranded adeno-associated virus
*Nrl*	Neural retina leucine zipper
DHDDS	Dehydrodolichyl diphosphate synthase
shRNA	Short hairpin RNA
HA	Hemagglutinin
ERG	Electroretinogram
PGK–Neo	Phosphoglycerate kinase I promoter–neomycin phosphotransferase gene
FLP	Flippase recombinase
FRT	Flippase recognition target
PBS	Phosphate-buffered saline
RPE	Retinal pigment epithelium
OS	Outer segment
IS	Inner segment
ONL	Outer nuclear layer
OPL	Outer plexiform layer
INL	Inner nuclear layer
IPL	Inner plexiform layer
WT	Wild type
ORF	Open reading frame
UTR	Untranslated region
